# Watching eyes do not stop dogs stealing food: evidence against a general risk-aversion hypothesis for the watching-eye effect

**DOI:** 10.1038/s41598-020-58210-4

**Published:** 2020-01-24

**Authors:** Patrick Neilands, Rebecca Hassall, Frederique Derks, Amalia P. M. Bastos, Alex H. Taylor

**Affiliations:** 1 School of Psychology, University of Auckland, Auckland, 1010 New Zealand; 2 Groningen Institute for Evolutionary Life Sciences, GELIFES, University of Groningen, 9712 CP Groningen, The Netherlands

**Keywords:** Zoology, Animal behaviour, Psychology

## Abstract

The presence of pictures of eyes reduces antisocial behaviour in humans. It has been suggested that this ‘watching-eye’ effect is the result of a uniquely human sensitivity to reputation-management cues. However, an alternative explanation is that humans are less likely to carry out risky behaviour in general when they feel like they are being watched. This risk-aversion hypothesis predicts that other animals should also show the watching-eye effect because many animals behave more cautiously when being observed. Dogs are an ideal species to test between these hypotheses because they behave in a risk-averse manner when being watched and attend specifically to eyes when assessing humans’ attentional states. Here, we examined if dogs were slower to steal food in the presence of pictures of eyes compared to flowers. Dogs showed no difference in the latency to steal food between the two conditions. This finding shows that dogs are not sensitive to watching-eyes and is not consistent with a risk-aversion hypothesis for the watching-eye effect.

## Introduction

Recent work has suggested that humans alter their behaviour when they know they are being observed^1–8^. Strikingly, this tendency appears to extend to humans’ behaviour when merely being in the presence of eye images. For example, in lab studies where participants play economic games, people appear to donate more generously in the presence of images of eyes^9–11^, even when the images are as minimal as three dots arranged as an inverted triangle^12^. This watching-eye effect also appears to generalize to the field. People display a tendency to donate more money to charity or an honesty box when there is an image of eyes on the boxes or solicitation materials compared control images^13–15^, and they appear to be less likely to litter when there are posters with eyes on them in the surrounding environment^16,17^.

While there have been concerns about the effect not being robust^18,19^, a recent meta-analysis showed that watching-eyes results in a robust reduction in anti-social behaviour^20^. Variation in effect size between studies appears to be dependent on the degree to which subjects attend to eyes^21^, and on subjects being in situations where being watched might have real-world consequences (e.g. when subjects are not in environments where there is already a high chance they are being watched^22^, or where they are likely to be anonymous^18,23–25^). Crucially, if the watching-eye effect was simply due to human-related images reinforcing social norms or making people feel guilty, any images relating to the human body should produce the same effect and the magnitude of the effect would be the same whether the subject’s actions are public or not. However, instead, it has been shown that images of other body parts do not induce the watching-eye effect and the magnitude of the effect is reduced when subjects’ actions are anonymous^21^. This suggests that the monitoring aspect of eyes is crucial for explaining the watching-eye effect.

Whilst the extent to which societies engage in punishment varies, the universality of punishment in humans^26,27^ is striking in comparison to its rarity in other animals^28–30^. It has been argued that third-party punishment, where an observer punished an individual for actions directed towards another person, has evolved in humans to enable large scale cooperation^31–35^. This has lead to the claim that the watching-eye effect is a by-product of species-specific adaptations in humans relating to reputation-management^10,20,21,36^. This hypothesis posits that humans are highly sensitive to any cues of being watched in order to avoid being observed breaking social rules and so preserve their good reputations and avoid punishment.

However, while wide-scale third-party punishment might be unique to humans, reputation management is not the only context in which being watched matters. Rather than being related to reputation management *per se*, the watching-eye effect may reflect a more general risk-aversion strategy: individuals simply act more cautiously when they feel they are being watched because many actions, including breaking social rules, are riskier when being observed. In support of this hypothesis, a tendency to act more cautiously in the presence of eyes is prevalent across the animal kingdom. For example, the eye spots on caterpillars and other prey species exploit their would-be predators’ sensitivity to cues of being observed^37–39^, and birds such as herring gulls are slower to approach food when a human is looking at them^40^. Animals may also engage in tactical deception by altering their behaviour when being watched by more dominant individuals so as to avoid being attacked for taking food or engaging in reproductive activity^41–44^. Support for this ‘risk-aversion’ hypothesis also comes from work on the watching-eye effect in humans, as people who score highly on risk-aversion measures tend to show greater susceptibility to the watching-eye effect^45^. In contrast, inter-personal sensitivity, which relates to reputation-management, does not predict such susceptibility^45^. Rather than simply assuming that the watching-eye effect reflects human-specific adaptations, it is important to rule out alternative evolutionary explanations such as it being a by-product of general gaze aversion^46^.

Cross-species comparisons are potentially a powerful way to distinguish between the reputation-management and risk-aversion hypotheses. If this effect is the result of a general tendency to act more cautiously while being watched, we would predict that other animal species should also show this effect. In contrast, if the watching-eye effect is the result of human-specific adaptations relating to reputation management, we would predict that the watching-eye effect should be unique to humans. To date, outside of humans, the watching-eye effect has only been explored in chimpanzees, who do not react to images of eyes^36^. However, chimpanzees may be a poor model species for testing between these hypotheses because they do not attend specifically to eyes as cues of visual attention^47^. While they can engage in gaze-following, where they follow gazes round barriers, and preferentially beg from humans visually attending to them, they primarily rely on head and body orientation over eye orientation to do so^48,49^. This reliance on body and head orientation reflects the fact that, like most primates, the sclera of chimpanzees’ eyes are dark and “camouflaged”, making eyes less salient as cues of visual attention^50^. Humans differ from other primates in having white, conspicuous sclera and being highly sensitive to eyes as cues of visual attention and the “cooperative eye” hypothesis posits that this is because eye contact plays a key role in facilitating cooperative interactions amongst humans^47^. As such, rather than refuting the risk-aversion hypothesis, the lack of watching-eye effect in chimpanzees may simply be a result of eyes not being salient cues of visual attention to chimpanzees.

This lack of sensitivity towards eyes as cues of visual attention may not be restricted to chimpanzees. While other animals^51^, particularly birds, have been shown to use eye direction as a cue of visual attention^52–55^, they appear to use this cue in limited contexts. For example, ravens appear to follow human gazes around barriers^56^ but do not use the gaze of either humans^57^ or informed conspecifics^58^ to locate hidden food. Similarly, monkeys are more likely to steal from an experimenter whose eyes are covered compared to an experimenter whose eyes are visible^59^ but do not appear to use eye gaze as cues in either object-choice tasks^60^ or for choosing which experimenter to approach in order to beg for food^61^. As such, refuting the risk-aversion hypothesis is not trivial because the absence of the watching-eye effect in other animals may simply reflect that many species appear not to find eyes to be highly salient cues of visual attention compared to other cues such as head or body orientation.

In contrast, dogs are an excellent model species for testing between the risk-aversion and reputation-management hypotheses. Similarly to chimpanzees^62^, dogs appear to alter their behaviour in order to avoid direct punishment when being observed^63,64^ but, unlike chimpanzees, are highly sensitive to eyes as cues of visual attention^65^. Dogs use eye contact as a cue of visual attention in a range of contexts including assessing whether to steal food^63,64^, gaze-following around barriers^66^, and deciding which human to approach in begging paradigms^64,67^. Additionally, dogs use eye contact as a means to establish both communicative intent^68,69^, and social bonds^70^. In contrast, chimpanzees do not successfully use eye contact as a means of establishing communicative intent^71^, and while mutual gaze does play a role in mother-infant bonding in chimpanzees, gazes tend to be shorter in duration and mutual gaze is rare outside of the mother-infant pairing^50,72^. A similar reliance on eye contact is not found in wolves^70,73,74^, underlining dogs’ unparalleled sensitivity to attention to eye contact as a means of communication^47,50^. These findings mean that any failure to find the watching-eye effect in dogs cannot be attributed to dogs not attending specifically to eyes. As such, dogs are an ideal model species for testing whether risk-aversion or reputation management generates the watching eye effect. If this effect is the result of a general tendency to reduce risk-taking behaviour when an individual feels watched, dogs should also behave less anti-socially in the presence of pictures of eyes. In contrast, if the watching-eye effect is the result of human-specific adaptations for reputation management, images of eyes should have no effect on dog behaviour.

To test between these hypotheses, we presented dogs with a food-stealing experiment consisting of two trials: a baseline ‘Go’ trial, where the owner encouraged the dog to take food which had been placed on the ground, and a test ‘Leave’ trial, where the owner forbade the dog from taking the food. In both trials, the owner turned their back after giving the command and a photo was revealed above the food. For half of the dogs, the revealed photo was of eyes and for the other half, it was of flowers. We then compared the approach speed to the food when dogs had been commanded to either take the food or leave it. If dogs experience the watching-eye effect, we predicted dogs in the Eye condition would approach the food slower in the ‘Leave’ trial than dogs in the Flower condition. In contrast, if eyes were a generally aversive stimulus for dogs, we predicted dogs in the Eye condition would approach the food slower in both the ‘Go’ trial and the ‘Leave’ trial.

## Results

Using Trial Type (‘Go’ trial vs ‘Leave’ trial) and Condition (Eyes condition vs Flowers condition) as fixed effects, and participant as a random effect, we constructed several mixed-effects Bayesian ANOVA models. The best fitting model was the Trial Type-only model (Bayesian Mixed Effect model: BF = 6.41 × 10^8^; see Table S1 for all model details) and including Trial Type overwhelmingly increases the model fit (BF_incl_ = 6.44 × 10^8^). This suggests that dogs understood the command, taking much longer to steal food in the ‘Leave’ trials than in the ‘Go’ trials. If dogs found the images of eyes more aversive in general, including condition in the model should improve model fit but instead including it substantially reduced model fit (BF_incl_ = 0.230). Crucially, including the Condition*Trial Type interaction also substantially decreased model fit (BF_incl_ = 0.271), suggesting that dogs in the Eyes condition did not take longer to steal food during ‘Leave’ trials compared to dogs in the Flower condition.

As previous research has suggested that the extent to which humans attend to eyes affects the likelihood that they will show the watching-eye effect^21^, we re-ran this analysis but controlled for the proportion of the time that the dogs looked at the photo during the trial. Results from this analysis were qualitatively the same as the original analysis. The Trial Type-only model remained the best fitting model (Bayesian Mixed Effect model: BF = 7.38 × 10^8^;see Table S2 for all model details) and including Trial Type overwhelmingly increases the model fit (BF_incl_ = 8.82 × 10^5^). Similarly to our previous analysis, including either Condition (BF_incl_ = 0.350) or Condition*Trial Type (BF_incl_ = 0.290) interaction reduces the fit of the model.

**Figure 1:**
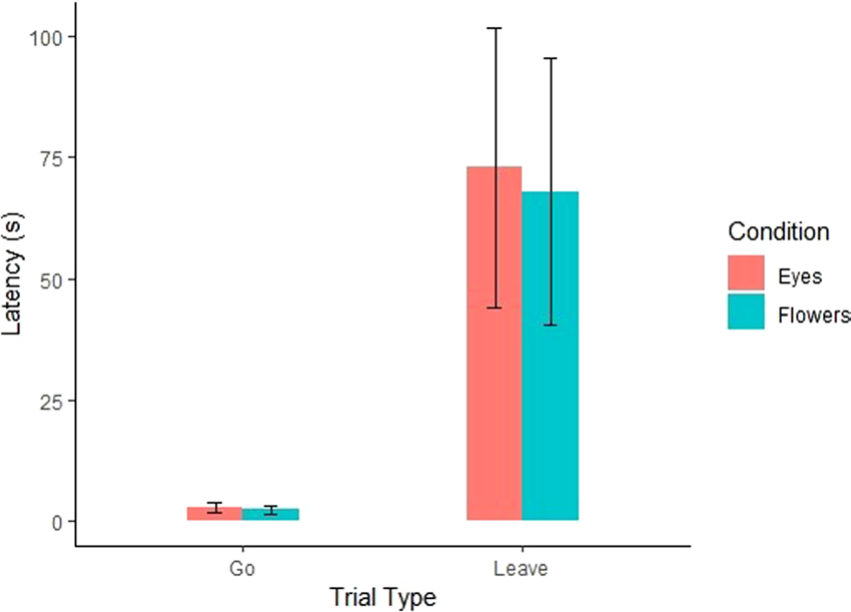
Dogs are sensitive to their owners’ commands but do not show the watching-eye effect. Dogs were slower to approach the food (Bayesian Mixed Effect ANOVA: Trial Type BF_incl_ = 6.44 × 10^8^) in the leave trials (Leave Eye trials: x ± 95% CI: 72.94 ± 20.34 s; Leave Flower trials x ± 95% CI: 67.97 ± 17.49 s) than in the go trials (Go Eye trials x ± 95% CI: 2.93 ± 0.723 s; Go Flower trials x ± 95% CI: 2.36 ± 0.507 s), suggesting that they understood the command. However, in the leave trials, dogs in the eyes condition were no slower to approach the food than dogs in the flowers condition (Bayesian Mixed Effect ANOVA: Trial Type*Condition BF_incl_ = 0.271) and as such did not demonstrate the watching-eyes effect.

Additionally, in order to specifically get at our comparison of interest, we compared the ‘Leave’ latency in both conditions after adjusting for differences in individual dogs’ approach speed. This adjustment was made by subtracting the ‘Go’ latency from the ‘Leave’. If the dogs display the watching-eye effect, we would predict that the adjusted latency would be higher in the eyes condition than in the flowers condition. However, when comparing the adjusted latencies in the ‘Leave’ trials, we continue to find substantial support for the null hypothesis: there was no difference (Bayesian Independent-Samples t-test, BF = 0.316) in the adjusted latency with which the dogs in the Eye condition (x ± 95% CI: 70.01 ± 19.70 s) stole food compared to the dogs in the Flower condition (65.61 ± 18.56 s). Both sets of analyses, therefore, suggest that images of eyes have no effect on how quickly dogs approach forbidden food, despite the dogs clearly understanding the command to leave the food (Fig. 1).

## Discussion

Our results show that dogs were no slower to approach forbidden food in the presence of images of eyes compared to images of flowers. Thus, unlike humans^20^, but like chimpanzees^36^, pictures of eyes do not reduce the frequency of anti-social behaviour in dogs. This lack of effect cannot be explained simply as being the result of the dogs not understanding that they were forbidden from taking the food. Dogs showed a clear understanding of the social rule given by their owner, being substantially slower to take food in the ‘Leave’ trials than in the ‘Go’ trials. Furthermore, as noted in the introduction, dogs attend closely to eyes across a range of contexts. Therefore, unlike in chimpanzees, this lack of effect cannot be attributed to the lack of salience of eyes as cues of visual attention in dogs. Additionally, this lack of effect cannot be attributed to dogs not recognizing pictures of eyes. Dogs recognize pictures of human faces^75,76^ and appear to use similar neural mechanisms to process these images as humans^77,78^. Furthermore, dogs show a preference for attending to eyes in these pictures^79^ and can discriminate between faces and emotions even when presented with partial images of faces or isolated images of eyes^80–82^. As such, the fact that dogs do not show the watching-eye effect is not consistent with the risk-aversion hypothesis. Despite the fact that dogs show risk aversion when being watched by actual humans in similar forbidden food paradigms^63^, the presence of pictures of eyes has no effect on how cautiously dogs approach food after being commanded to leave it.

These results suggests that the watching-eye effect cannot be explained in terms of the general tendency of animals to act more cautiously when being observed. Otherwise, we would predict that dogs in our study and chimpanzees in a previous study^36^ should also show the watching-eye effect. Similarly, these results suggest that the watching-eye effect is not the result of social living, where there may be pressure to be sensitive to gaze to avoid competition with, and potential aggression, from dominant group members^42^. Again, if this was the case, we would predict that dogs and chimpanzees, both social species, would show the watching-eye effect. Rather, while much work remains to be done to rule out non-adaptative explanations^46^, these results are consistent with the hypothesis that the watching-eye effect is a by-product of adaptations relating to reputation and the need to avoid punishment in humans^21,35,36^, though it remains to be determined whether this itself is due to the evolution of cognitive mechanisms specific to reputation-management or an increased sensitivity in the mechanisms that humans share with animals that regulate gaze sensitivity^32^.

Crucially, while dogs understand social rules, such as being slower to steal food when commanded to leave it in our study, this understanding appears to be specific to the person giving the rule and does not generalize to novel observers. For example, dogs are more likely to steal forbidden food when a novel observer replaces the person giving the command^83^. As such, in our study, dogs may not have generalised the social rule of not taking food established by their owner to other observers, and so did not react to the watching-eye stimulus. In contrast, generalizing a rule from a specific situation to novel situations is an important precursor for the development of social norms in children^84^. Such social norms play a key role in cooperation and reputation-management^85^ and following these norms is crucial to avoid punishment^86^. Therefore, a tendency to generalize social rules across individuals (i.e. develop social norms) as a means to avoid costly punishment^36^ may have created the selection pressure for the watching-eye effect. In contrast, dogs’ tendency to anchor social rules to specific people^83^ could be a key explanation as to why dogs appear to be sensitive to social rules but do not show the watching-eye effect. Strikingly, chimpanzees also do not generalize social rules; being no less likely to steal food when a third-party is watching^87^.

While it has been widely assumed that the watching-eye effect is evidence of the key role reputation-management plays in explaining the complexity of human social structures^2,21,36,88,89^, this has been left largely untested. Our findings suggest that general risk aversion is not a key driver of the watching eye effect and is consistent with the claims that the effect is indeed the result of adaptations relating to reputation-management. However, further work is clearly required to confirm this, both with other species and experimental paradigms.

We tested dogs in an analogous situation to previous studies where humans had to decide to conform to either explicit^9–12^ or implicit^13–17^ social rules while in the presence or absence of eye cues. However, it is not currently clear how humans react in the absence of such a rule. Future work repeating this experiment in the absence of social rules, both in humans and dogs would be an interesting line of inquiry.

Similarly, the ontogeny of the watching-eye effect in humans remains underexplored and this makes it difficult to assess the role of the development of social norm sensitivity in producing the watching-eye effect. Two studies have found no evidence for the watching-eye effect in children^90,91^. However, a third study, which used real photos of eyes rather than stylized images and primed children with the test stimuli prior to the experiment, did find evidence of the watching-eye effect in pre-schoolers^92^. The authors posited that without the priming, the infants did not attend to the eyes during the experiment. As well as leaving the picture of the ontogeny of the watching-eye effect unclear, these results leave open the possibility that dogs may show the watching-eye effect under a modified experimental paradigm even if they do not show it with the standard procedure. However, dogs in our study attended to the images for 30% of the trial on average and the amount of time that the dogs attended to the image had no impact on how quickly the dogs stole the food. As such, the lack of attention to stimuli seems to be an unlikely explanation for why dogs do not show the watching-eye effect.

Finally, while it has been assumed that the watching-eye effect is unique to humans^21,89,93^, there has been a paucity of research into the watching-eye effect outside of humans. This lack of cross-species comparisons has hampered abilities to draw conclusions about the specificity of the watching-eye effect. Testing for the watching-eye effect in animals, such as cleaner wrasse, which engage in rudimentary reputation management through techniques such as image scoring^94^, would be a useful way to determine whether this effect is indeed unique to humans or whether it also generalizes to other animals which engage in more basic forms of reputation-management.

In conclusion, our findings suggest that dogs, despite being highly sensitive to human eyes, do not show the watching-eye effect in a testing paradigm analogous to the standard paradigm used to investigate the watching-eye effect in humans. Alongside previous research in chimpanzees^36^, our findings suggest that the watching-eye effect cannot be explained in terms of the general gaze aversion found across the animal kingdom^51^. However, further research is required to explore whether the watching-eye effect can be found under alternative testing conditions and with a wider range of species before any conclusions can be strongly drawn that the watching-eye effect is a human-specific phenomenon related to reputation-management.

## Methods

### Ethics statement

The present study was approved by the University of Auckland Animal Ethics Committee R001826 and the University of Auckland Human Ethics Committee R018410. All work with the dogs was in accordance with the guidelines of the New Zealand National Animals Ethics Advisory Committee. Dogs were recruited through owners’ responses to online applications. Written informed consent for participating in this study was obtained from the owners.

### Participants

A total of 58 dogs were recruited. Our sample size was determined by a stopping rule where we included dogs until we obtained a BF of > 3 or < 0.333 and had at least 15 dogs in each condition. All dogs were pet dogs (aged 2–10 years old) which were accompanied to the lab by their owners (see Supplementary Table S3 for details for dogs included in the study). In order to participate in the study, dogs had to meet two criteria: i) they had to leave food for at least 5 s after their owner had told them to do so before turning their back, and ii) after three attempts, they had to be willing to approach the food within 5 s of the owner telling the dog to take the food and then turning their back. This excluded dogs that that were not trained to leave food or were too cautious to approach food in a novel environment. Participants took part either in the eye condition or the flower condition.

### Set up of room

**Figure 2:**
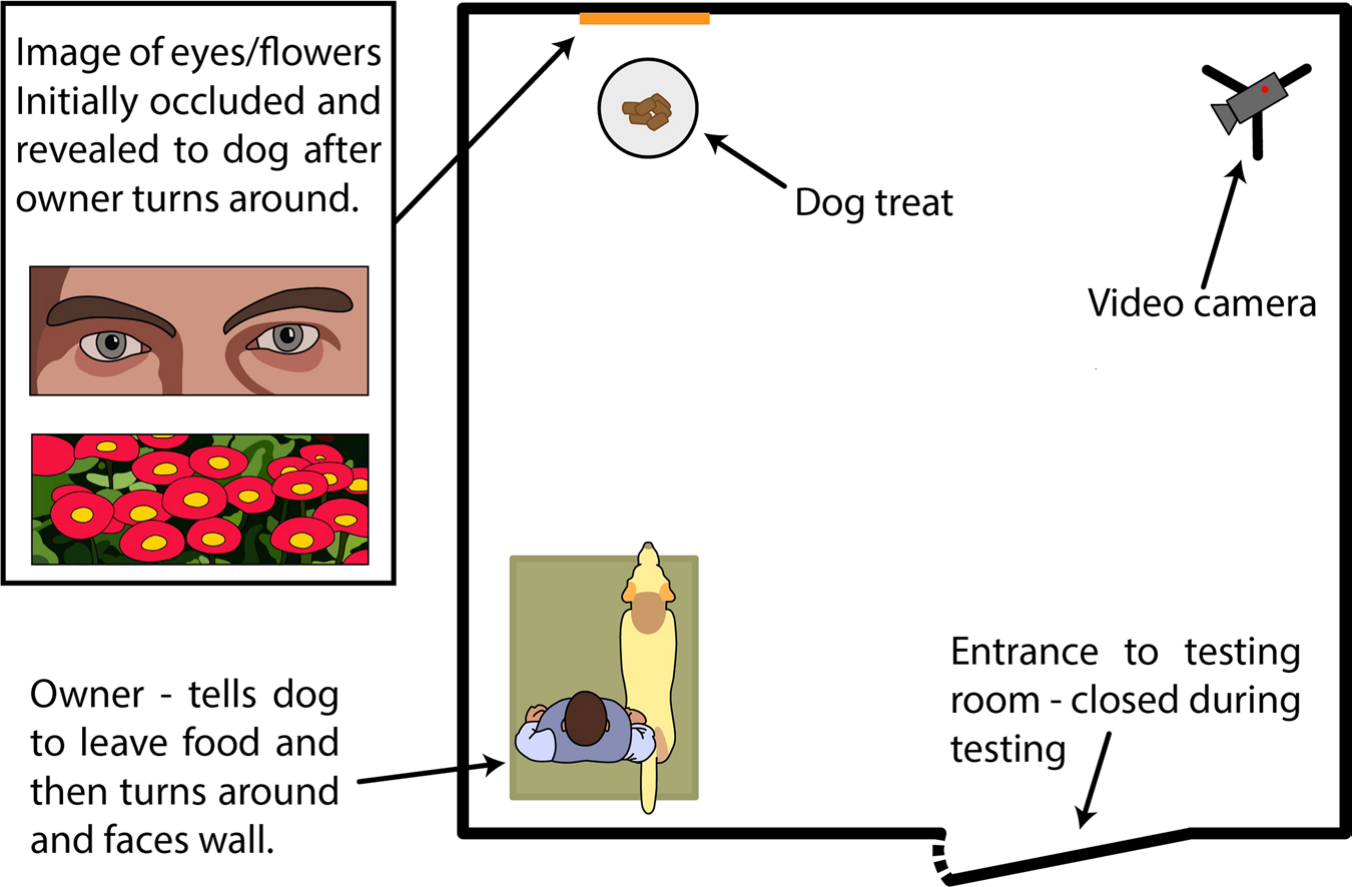
Set up of experimental room. After the dog was settled in the room (3.4 × 3.6 m), the owner took the dog off the lead, instructed it to either take or leave the food (depending on trial type) and then turned to face the wall. After the owner turned around, an experimenter in an adjacent room moved a cardboard barrier across, revealing a picture of either eyes or flowers.

The experiment took part in a dedicated testing room (3.6 m × 3.4 m). Dogs were settled on a dog bed opposite a cardboard barrier 3.4 m away from the dog. After the owner turned their back, the cardboard was slid to one side by a hidden experimenter pulling a fishing line attached to the cardboard, revealing the picture (see Fig. 2 for set up). Two sets of ‘eyes’ pictures and ‘flower’ pictures were used (Supplementary Fig. S1).

### Protocol

Dogs took part in two trials. In both trials, the owner would give the dog a command before turning their back. After the owner had turned their back, the cardboard barrier was slid across to reveal the picture behind it. A trial lasted until either the dog had taken the food or after three minutes had elapsed. At the end of each trial, the experimenter would re-enter the room and slide the cardboard over the picture again. Only at that point would owners be asked to turn back around. The first trial was a baseline ‘Go’ trial where the owner encouraged the dog to take the food and the second trial was the test ‘Leave’ trial where the owner commanded the dog to leave the food. Owners were told to use the same release and leave commands as they would at home. Dogs were exposed to either a photo of eyes or a photo of flowers. Ideally, dogs would have been exposed to both images. However, when this study was piloted with a within-study design, dogs had learnt they could approach the food with impunity by the fourth trial and so the large order effects outweighed the benefits of increased statistical power. As such, we used a between-subjects design for this current study.

### Analysis

The latency to approach the food was recorded in both the ‘Go’ and ‘Leave’ trials. Latency was timed from the point that the owner gave the command until the dog had eaten the food. An additional coder, blind to condition, coded the approach latency for 40% of the sample. The high intra-class correlation (ICC = 0.99) indicates excellent levels of agreement between coders. To analyse the data, we constructed several mixed-effects Bayesian ANOVA models. The factors included in these models were Trial Type (Leave vs Go), Condition (Eye vs Flower), and a Trial Type*Condition interaction. Due to the repeated-measures aspect of the design (all dogs took part in both a ‘Go’ and ‘Leave’ trial), participant was included as a random effect in all models. Each model was compared to a null model, which only contained participant as a random effect. Additionally, an analysis of effects was carried out to determine the inclusion BF for each individual factor. Inclusion BFs are calculated by comparing the fit of models containing the factor against the fit of models not containing that factor. BF_incl_ > 3 indicate that including a factor substantially increases model fit while BF_incl_ < 0.333 indicates a factor substantially decreases model fit. Each model was constructed with objective priors of prior width (r) = 1 for fixed effects and r = 0.5 for random effects.

As the extent to which humans attended to images of eyes appeared to affect their likelihood of showing the watching-eye effect^21^, we re-ran this analysis but included the proportion of time that the dogs looked at the picture as a covariate for each model. Each model was compared to a null model which contained participant as a random effect and proportion of time looking at the picture as a covariate. Again, models were constructed with objective priors of r = 1 for fixed effects and r = 0.5 for random effects.

Additionally, in order to specifically get at our comparison of interest, we compared the ‘Leave’ latency in both conditions after adjusting for differences in individual dogs’ approach speed. This adjustment was made by subtracting the ‘Go’ latency from the ‘Leave’. If the dogs display the watching-eye effect, we would predict that the adjusted latency would be higher in the eyes condition than in the flowers condition. Comparisons between the adjusted ‘Leave’ latencies were analysed using a Bayesian independent-samples t-test. The prior distribution for the alternative hypothesis was a Cauchy half-distribution, centred on an effect size of 0, with r = 0.707. All analyses were carried out using JASP 0.10.0.0 (JASP team, 2019.) This study design was pre-registered (http://aspredicted.org/blind.php?x=j6er8v). It should be noted using the Go trial as a baseline to adjust the dogs’ Leave latencies meant it was necessary to have the owners give the ‘Go’ command on the same trial. Whilst this means that it is impossible to fully disentangle the effect of the command on the dogs’ latency to approach food from order effect, we concluded that the extreme implausibility that dogs would approach food slower on a 2^nd^ trial after being able to take it without punishment in the previous trials made this a worthwhile trade-off.

## Supplementary information

Supplementary Information.

Supplementary Information 2.

## Data Availability

The data associated with this research is available in the supplementary materials accompanying this manuscript.

## References

[1] Engelmann D, Fischbacher U (2009). Indirect Reciprocity and Strategic Reputation Building in an Experimental Helping Game. Games Econ. Behav..

[2] Burton-Chellew, M. N., El Mouden, C. & West, S. A. Evidence for strategic cooperation in humans. *Proc. R. Soc. B Biol. Sci*. **284**, (2017). 10.1098/rspb.2017.0689 PMC5474078 28592673

[3] Cañigueral R, Hamilton AFdC (2019). Being watched: Effects of an audience on eye gaze and prosocial behaviour. Acta Psychol. (Amst)..

[4] Bereczkei T, Birkas B, Kerekes Z (2007). Public charity offer as a proximate factor of evolved reputation-building strategy: an experimental analysis of a real-life situation. Evol. Hum. Behav..

[5] Soetevent AR (2005). Anonymity in giving in a natural context - A field experiment in 30 churches. J. Public Econ..

[6] Satow KL (1975). Social approval and helping. J. Exp. Soc. Psychol..

[7] Filiz-Ozbay E, Ozbay EY (2014). Effect of an audience in public goods provision. Exp. Econ..

[8] Sylwester K, Roberts G (2010). Cooperators benefit through reputation-based partner choice in economic games. Biol. Lett..

[9] Oda R, Niwa Y, Honma A, Hiraishi K (2011). An eye-like painting enhances the expectation of a good reputation. Evol. Hum. Behav..

[10] Burnham TC, Hare B (2007). Engineering Human Cooperation Does Involuntary Neural Activation Increase Public. Hum. Nat..

[11] Haley KJ, Fessler DMT (2005). Nobody’s watching? Subtle cues affect generosity in an anonymous economic game. Evol. Hum. Behav..

[12] Rigdon M, Ishii K, Watabe M, Kitayama S (2009). Minimal social cues in the dictator game. J. Econ. Psychol..

[13] Bateson M, Nettle D, Roberts G (2006). Cues of being watched enhance cooperation in a real-world setting. Biol. Lett..

[14] Krupka EL, Croson RTA (2016). The differential impact of social norms cues on charitable contributions. J. Econ. Behav. Organ..

[15] Ekström M (2011). Do Watching Eyes Affect Charitable Giving? Evidence from a Field Experiment. Exp. Econ..

[16] Ernest-jones M, Nettle D, Bateson M (2011). Effects of eye images on everyday cooperative behavior: a field experiment. Evol. Hum. Behav..

[17] Bateson M, Callow L, Holmes JR, Redmond Roche ML, Nettle D (2013). Do images of ‘watching eyes’ induce behaviour that is more pro-social or more normative? A field experiment on littering. PLoS One.

[18] Fehr, E. & Schneider, F. Eyes are on us, but nobody cares: Are eye cues relevant for strong reciprocity? *Proc. R. Soc. B* **277**, (2010). 10.1098/rspb.2009.1900 PMC2871936 20031986

[19] Northover SB, Pedersen WC, Cohen AB, Andrews PW (2017). Artificial surveillance cues do not increase generosity: two meta-analyses. Evol. Hum. Behav..

[20] Dear K, Dutton K, Fox E (2019). Do ‘watching eyes’ influence antisocial behavior? A systematic review & meta-analysis. Evol. Hum. Behav..

[21] Vaish A, Kelsey CM, Tripathi A, Grossmann T (2017). Attentiveness to eyes predicts generosity in a reputation-relevant context. Evol. Hum. Behav..

[22] Powell KL, Roberts G, Nettle D (2012). Eye Images Increase Charitable Donations: Evidence From an Opportunistic Field Experiment in a Supermarket. Ethology.

[23] Saunders, T. J., Taylor, A. H. & Atkinson, Q. D. No evidence that a range of artificial monitoring cues influence online donations to charity in an MTurk sample. *R. Soc. Open Sci*. **3**, (2016). 10.1098/rsos.150710 PMC5098958 27853533

[24] Raihani, N. J. & Bshary, R. A positive effect of flowers rather than eye images in a large-scale, cross-cultural dictator game. *Proc. R. Soc. B* **279**, (2012). 10.1098/rspb.2012.0758 PMC3396908 22673357

[25] Tane K, Takezawa M (2011). Perception of human face does not induce cooperation in darkness. Lett. Evol. Behav. Sci..

[26] Henrich J (2006). Costly punishment across human societies. Science..

[27] Marlowe FW (2008). More ‘altruistic’ punishment in larger societies. Proc. R. Soc. B Biol. Sci..

[28] Raihani NJ, Thornton A, Bshary R (2012). Punishment and cooperation in nature. Trends Ecol. Evol..

[29] Riehl C, Frederickson ME (2016). Cheating and punishment in cooperative animal societies. Phil Trans R Soc B.

[30] Riedl K, Jensen K, Call J, Tomasello M (2012). No third-party punishment in chimpanzees. Proc. Natl. Acad. Sci. USA.

[31] Melis AP, Semmann D (2010). How is human cooperation different?. Philos. Trans. R. Soc. B Biol. Sci..

[32] Buckholtz JW, Marois R (2012). The roots of modern justice: Cognitive and neural foundations of social norms and their enforcement. Nat. Neurosci..

[33] Fehr E, Fischbacher U (2004). Third-party punishment and social norms. Evol. Hum. Behav..

[34] Hauser M, McAuliffe K, Blake PR (2009). Evolving the ingredients for reciprocity and spite. Philos. Trans. R. Soc. B Biol. Sci..

[35] Jensen K (2010). Punishment and spite, the dark side of cooperation. Philos. Trans. R. Soc. B Biol. Sci..

[36] Nettle D, Cronin KA, Bateson M (2013). Responses of chimpanzees to cues of conspecific observation. Anim. Behav..

[37] Janzen DH, Hallwachs W, Burns JM (2010). A tropical horde of counterfeit predator eyes. Proc. Natl. Acad. Sci..

[38] De Bona, S., Valkonen, J. K., López-Sepulcre, A. & Mappes, J. Predator mimicry, not conspicuousness, explains the efficacy of butterfly eyespots. *Proc. R. Soc. B Biol. Sci*. **282**, (2015). 10.1098/rspb.2015.0202 PMC4426626 25854889

[39] Kjernsmo K, Merilaita S (2017). Resemblance to the Enemy’s Eyes Underlies the Intimidating Effect of Eyespots. Am. Nat..

[40] Goumas M, Burns I, Kelley LA, Boogert NJ (2019). Herring gulls respond to human gaze direction. Biol. Lett..

[41] Hall K, Brosnan SF (2017). Cooperation and deception in primates. Infant Behav. Dev..

[42] Whiten A, Byrne RW (1988). Tactical deception in primates. Behav. Brain Sci..

[43] Bugnyar T, Heinrich B (2006). Pilfering ravens, Corvus corax, adjust their behaviour to social context and identity of competitors. Anim. Cogn..

[44] Holekamp KE, Sakai ST, Lundrigan BL (2007). Social intelligence in the spotted hyena (Crocuta crocuta). Philos. Trans. R. Soc. B Biol. Sci..

[45] Keller J, Pfattheicher S (2011). Vigilant Self-Regulation, Cues of Being Watched and Cooperativeness. Eur. J. Pers..

[46] Lloyd EA (2015). Adaptationism and the Logic of Research Questions: How to Think Clearly About Evolutionary Causes. Biol. Theory.

[47] Tomasello M, Hare B, Lehmann H, Call J (2007). Reliance on head versus eyes in the gaze following of great apes and human infants: the cooperative eye hypothesis. J. Hum. Evol..

[48] Bräuer J, Call J, Tomasello M (2005). All great ape species follow gaze to distant locations and around barriers. J. Comp. Psychol..

[49] Kaminski, J., Call, J. & Tomasello, M. Body orientation and face orientation: two factors controlling apes’ begging behavior from humans. **7**, 216–223 (2004). 10.1007/s10071-004-0214-2 15034765

[50] Kobayashi H, Hashiya K (2011). The gaze that grooms: Contribution of social factors to the evolution of primate eye morphology. Evol. Hum. Behav..

[51] Davidson GL, Clayton NS (2016). New perspectives in gaze sensitivity research. Learn. Behav..

[52] von Bayern AMP, Emery NJ (2009). Jackdaws Respond to Human Attentional States and Communicative Cues in Different Contexts. Curr. Biol..

[53] Carter J, Lyons NJ, Cole HL, Goldsmith AR (2008). Subtle cues of predation risk: Starlings respond to a predator’s direction of eye-gaze. Proc. R. Soc. B Biol. Sci..

[54] Clucas B, Marzluff JM, Mackovjak D, Palmquist I (2013). Do American Crows Pay Attention to Human Gaze and Facial Expressions?. Ethology.

[55] Garland A, Low J, Armstrong N, Burns KC (2014). Wild robins (Petroica longipes) respond to human gaze. Anim. Cogn..

[56] Bugnyar T, Stöwe M, Heinrich B (2004). Ravens, Corvus corax, follow gaze direction of humans around obstacles. Proc. R. Soc. B Biol. Sci..

[57] Schloegl C, Kotrschal K, Bugnyar T (2008). Do common ravens (Corvus corax) rely on human or conspecific gaze cues to detect hidden food?. Anim. Cogn..

[58] Schloegl C, Kotrschal K, Bugnyar T (2008). Modifying the object-choice task: Is the way you look important for ravens? *Behav*. Processes.

[59] Flombaum JI, Santos LR (2005). Rheus Monkeys Attribute Perceptions to Others. Curr. Biol..

[60] Anderson JR, Montant M, Schmitt D (1996). Rhesus monkeys fail to use gaze direction as an experimenter-given cue in an object-choice task. Behav. Processes.

[61] Maille, A., Engelhart, L., Bourjade, M. & Blois-Heulin, C. To beg, or not to beg? that is the question: Mangabeys modify their production of requesting gestures in response to human’s attentional states. *PLoS One* **7**, (2012). 10.1371/journal.pone.0041197 PMC3399851 22815969

[62] Hare B, Call J, Tomasello M (2001). Do chimpanzees know what conspecifics know?. Anim. Behav..

[63] Call J, Bräuer J, Kaminski J, Tomasello M (2003). Domestic dogs (Canis familiaris) are sensitive to the attentional state of humans. J. Comp. Psychol..

[64] Virányi Z, Topál J, Gácsi M, Miklósi Á, Csányi V (2004). Dogs respond appropriately to cues of humans’ attentional focus. Behav. Processes.

[65] Kaminski J, Nitzschner M (2013). Do dogs get the point? A review of dog-human communication ability. Learn. Motiv..

[66] Bräuer J, Call J, Tomasello M (2004). Visual perspective taking in dogs (Canis familiaris) in the presence of barriers. Appl. Anim. Behav. Sci..

[67] Gácsi M, Miklód Á, Varga O, Topál J, Csányi V (2004). Are readers of our face readers of our minds? Dogs (Canis familiaris) show situation-dependent recognition of human’s attention. Anim. Cogn..

[68] Kaminski J, Schulz L, Tomasello M (2012). How dogs know when communication is intended for them. Dev. Sci..

[69] Téglás E, Gergely A, Kupán K, Miklósi Á, Topál J (2012). Dogs’ Gaze Following Is Tuned to Human Communicative Signals. Curr. Biol..

[70] Nagasawa M (2015). Oxytocin-gaze positive loop and the coevolution of human-dog bonds. Science (80-.)..

[71] Kano F (2018). Human ostensive signals do not enhance gaze following in chimpanzees, but do enhance object-oriented attention. Anim. Cogn..

[72] Bard KA (2005). Group differences in the mutual gaze of chimpanzees (Pan Troglodytes). Dev. Psychol..

[73] Gácsi M (2009). Explaining dog wolf differences in utilizing human pointing gestures: Selection for synergistic shifts in the development of some social skills. PLoS One.

[74] Miklósi Á (2003). A Simple Reason for a Big Difference: Wolves Do Not Look Back at Humans but Dogs Do. Curr. Biol..

[75] Somppi S, Törnqvist H, Hänninen L, Krause C, Vainio O (2012). Dogs do look at images: Eye tracking in canine cognition research. Anim. Cogn..

[76] Amadei E, Guo K, Meints K, Mills D (2007). Discrimination of human and dog faces and inversion responses in domestic dogs (Canis familiaris). Anim. Cogn..

[77] Cuaya LV, Hernández-Pérez R, Concha L (2016). Our faces in the dog’s brain: Functional imaging reveals temporal cortex activation during perception of human faces. PLoS One.

[78] Guo K, Meints K, Hall C, Hall S, Mills D (2009). Left gaze bias in humans, rhesus monkeys and domestic dogs. Anim. Cogn..

[79] Somppi S, Törnqvist H, Hänninen L, Krause CM, Vainio O (2014). How dogs scan familiar and inverted faces: An eye movement study. Anim. Cogn..

[80] Müller CA, Schmitt K, Barber ALA, Huber L (2015). Dogs can discriminate emotional expressions of human faces. Curr. Biol..

[81] Pitteri E, Mongillo P, Carnier P, Marinelli L, Huber L (2014). Part-based and configural processing of owner’s face in dogs. PLoS One.

[82] Huber L, Racca A, Scaf B, Virányi Z, Range F (2013). Discrimination of familiar human faces in dogs (Canis familiaris). Learn. Motiv..

[83] Hertel, A., Kaminski, J. & Tomasello, M. Generalize or personalize - Do dogs transfer an acquired rule to novel situations and persons? *PLoS One* **9**, (2014). 10.1371/journal.pone.0102666 PMC4100895 25029253

[84] Rakoczy H, Schmidt MFH (2013). The Early Ontogeny of Social Norms. Child Dev. Perspect..

[85] Fehr E, Schurtenberger I (2018). Normative foundations of human cooperation. Nat. Hum. Behav..

[86] Fehr, E. & Fischbacher, U. Social norms and human cooperation. *Trends Cogn. Sci*. **8**, (2004). 10.1016/j.tics.2004.02.007 15050515

[87] Engelmann JM, Herrmann E, Tomasello M (2012). Five-Year Olds, but Not Chimpanzees, Attempt to Manage Their Reputations. PLoS One.

[88] Rand DG, Nowak MA (2013). Human cooperation. Trends Cogn. Sci..

[89] Milinski Manfred (2016). Reputation, a universal currency for human social interactions. Philosophical Transactions of the Royal Society B: Biological Sciences.

[90] Fujii T, Takagishi H, Koizumi M, Okada H (2015). The effect of direct and indirect monitoring on generosity among preschoolers. Sci. Rep..

[91] Vogt S, Efferson C, Berger J, Fehr E (2015). Eye spots do not increase altruism in children. Evol. Hum. Behav..

[92] Kelsey C, Grossmann T, Vaish A (2018). Early reputation management: Three-year-old children are more generous following exposure to eyes. Front. Psychol..

[93] Izuma K (2012). The social neuroscience of reputation. Neurosci. Res..

[94] Bshary R, Grutter AS (2006). Image scoring and cooperation in a cleaner fish mutualism. Nature.

