# Supplementary material for: Watching eyes do not stop dogs stealing food: evidence against a general risk-aversion hypothesis for the watching-eye effect
Source: Sci Rep. 2020 Jan 24;10:1153. doi: 10.1038/s41598-020-58210-4 (PMC6981177; doi:10.1038/s41598-020-58210-4)
Supplement: Supplementary file 1 — Supplementary Information. [file 41598_2020_58210_MOESM1_ESM.docx]

Watching eyes do not stop dogs stealing food: evidence against a general risk-aversion hypothesis for the watching-eye effect.

Patrick Neilands^1*^, Rebecca Hassall^1^, Frederique Derks^2^, Amalia P M Bastos^1^, & Alex H Taylor^1^

^1^School of Psychology, University of Auckland, Auckland, 1010, New Zealand

^2^Groningen Institute for Evolutionary Life Sciences, GELIFES, University of Groningen, 9712 CP, Groningen, The Netherlands

*Corresponding author: pnei460@aucklanduni.ac.nz

*Supplementary Material:*


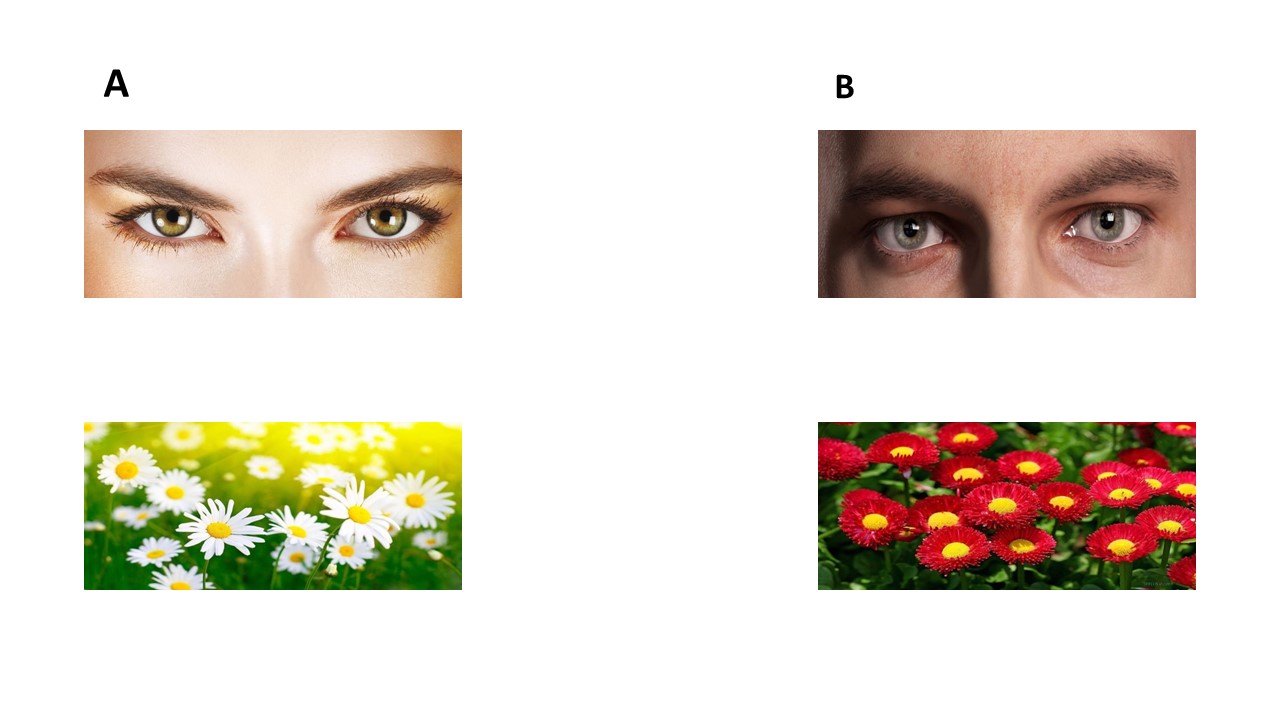


**Fig S1: Picture Sets for study.** A) Picture Set 1. B) Picture Set 2. All stimuli were 25cm x 11.1cm and were printed in the centre of an A4 page in the landscape orientation (29.7cm x 21.10cm.) Picture were initially occluded by a cardboard barrier and after the owner had given the command and turned their back, the barrier was removed, and the picture revealed to the dog.

**Table S1: Details of Bayesian mixed effect ANOVA models without controlling for proportion of looking time:** Four models were constructed: Trial Type-only (‘Leave’ vs ‘Go’ trials), Condition-only (‘Eyes’ picture vs ‘Flowers’), Condition + Trial Type, and Condition*Trial Type. Models were compared to a null (participant-only) model to see which model fitted the data best. Additionally, a analysis of effects was carried out by comparing the fit of models including a factor with models that didn’t include that factor. The Trial Type-only model was the best fitting model and Trial-Type was the only factor that substantially improved the fit of the model.

| *Model Comparison* | | |
| --- | --- | --- |
| **Model** | **BF_10_** | **Error %** |
| Null Model | 1.000 | - |
| Trial Type | 6.41 x 10^8^ | 1.87 |
| Condition | 0.23 | 0.995 |
| Condition + Trial Type | 1.47 x 10^8^ | 1.57 |
| Condition*Trial Type | 3.99 x 10^7^ | 1.94 |
| *Analysis of Effects* | | |
| **Effects** | **BF_incl_** |  |
| Trial Type | 6.44 x 10^8^ |  |
| Condition | 0.230 |  |
| Condition*Trial Type | 0.271 |  |

NB: All models include participant as a random effect.

**Table S2: Details of Bayesian mixed effect ANOVA models controlling for proportion of looking time:** Four models were constructed: Trial Type-only (‘Leave’ vs ‘Go’ trials), Condition-only (‘Eyes’ picture vs ‘Flowers’) , Condition + Trial Type, and Condition*Trial Type. All models controlled for the proportion of the time looking at the photo by including it as a fixed effect. Models were compared to a null (participant+ proportion looking time) model to see which model fitted the data best. Additionally, a analysis of effects was carried out by comparing the fit of models including a factor with models that didn’t include that factor. The Trial Type-only model was the best fitting model and Trial-Type was the only factor that substantially improved the fit of the model.

| *Model Comparison* | | |
| --- | --- | --- |
| **Model** | **BF_10_** | **Error %** |
| Null Model | 1.000 | - |
| Trial Type | 7.38 x 10^9^ | 8.01 |
| Condition | 0.23 | 10.03 |
| Condition + Trial Type | 1.74 x 10^8^ | 8.57 |
| Condition*Trial Type | 4.99 x 10^7^ | 8.98 |
| *Analysis of Effects* | | |
| **Effects** | **BF_incl_** |  |
| Trial Type | 8.82 x 10^5^ |  |
| Condition | 0.35 |  |
| Condition*Trial Type | 0.29 |  |

NB: All models include participant as a random effect and proportion looking time as a fixed effect.

**Table S3: Details for dogs taking part in study:** 58 dogs (female= 29) took part in this study. Half of them took part in the eyes condition (n=29) and the other half took part in the flowers condition (n=29). Dogs were between 2-10 years old.

| Dog | Age | Sex | Breed | Condition | Go Trial | Leave Trial | Differences | Picture Set |
| --- | --- | --- | --- | --- | --- | --- | --- | --- |
| Juve | 8 | F | Tibetan Terrier | Flowers | 3.02 | 1.628 | -1.392 | 1 |
| Poppy | 2 | F | Cocker Spaniel | Flowers | 1.163 | 2.953 | 1.79 | 1 |
| Spike | 2 | M | Boston Terrier | Eyes | 2.129 | 16.21 | 14.081 | 1 |
| Frankie | 3 | F | Fox Terrier X | Eyes | 1.64 | 3.149 | 1.509 | 1 |
| Ollie | 3 | M | Huntaway X | Flowers | 8.315 | 3.187 | -5.128 | 1 |
| Hobbes | 2 | M | Huntaway X | Eyes | 1.261 | 1.139 | -0.122 | 1 |
| Tobs | 2 | M | Huntaway/ Border Collie | Flowers | 1.95 | 180 | 178.05 | 1 |
| Milo | 9 | M | Labrador X | Eyes | 1.43 | 180 | 178.57 | 1 |
| Jet | 3 | F | Doberman | Flowers | 1.04 | 63 | 61.96 | 1 |
| Xanthe | 3 | F | Labrador | Eyes | 3.13 | 89.7 | 86.57 | 1 |
| Phoneix | 5 | F | Staffie/ Huntaway | Flowers | 1.424 | 44.61 | 43.186 | 1 |
| Willow | 2 | F | Border Collie | Eyes | 1.4 | 34.25 | 32.85 | 1 |
| Pippa | 2 | F | Ridgeback X | Flowers | 1.62 | 2.73 | 1.11 | 2 |
| Loki | 2 | M | Husky/ Border Collie | Eyes | 1.4 | 78.21 | 76.81 | 2 |
| Whizz | 2 | F | Labrador | Flowers | 1.17 | 1.78 | 0.61 | 2 |
| Winnie | 9 | F | Shar Pei | Eyes | 2.07 | 6.72 | 4.65 | 2 |
| Tui | 2 | F | Labrador | Flowers | 3.27 | 4.4 | 1.13 | 2 |
| Mawhai | 3 | M | Jadgterrir/  Border Terrier | Eyes | 1.53 | 13.13 | 11.6 | 2 |
| Grace | 7 | F | Boxer | Flowers | 2.44 | 95.99 | 93.55 | 2 |
| Wally | 2 | M | German Shorthaired Pointer X | Eyes | 1.08 | 180 | 178.92 | 2 |
| Luna | 4 | F | Staffie/ Australian Kelpie | Flowers | 1.04 | 1.737 | 0.697 | 2 |
| Louie | 3 | M | Labrador/ Staffie | Eyes | 1.169 | 3.099 | 1.93 | 2 |
| Monty | 3 | M | Huntaway/ Labrador | Flowers | 1.732 | 180 | 178.268 | 2 |
| Skater | 6 | F | Rottweiler/ English Mastiff | Eyes | 13.168 | 25.438 | 12.27 | 2 |
| Dice | 5 | M | Standard Poodle | Flowers | 1.503 | 6.592 | 5.089 | 2 |
| Lola | 3 | F | Labrador | Eyes | 1.481 | 180 | 178.519 | 2 |
| Mirka | 2 | F | Corgi | Flowers | 2.815 | 180 | 177.185 | 2 |
| Chewie | 3 | M | Boxer X | Eyes | 2.511 | 43.409 | 40.898 | 2 |
| Mack | 2 | M | Fox Terrier/ Poodle | Flowers | 1.488 | 131.655 | 130.167 | 1 |
| Brock | 3 | M | Rottweiler | Eyes | 1.734 | 180 | 178.266 | 1 |
| Princess Polar | 5 | F | Labrador X | Flowers | 1.24 | 180 | 178.76 | 1 |
| Charlie | 3 | M | Border Collie | Eyes | 2.236 | 180 | 177.764 | 1 |
| Dora | 9 | F | Boston Terrier | Flowers | 9.398 | 46.225 | 36.827 | 1 |
| Bronx | 4 | M | German Shepherd X | Eyes | 1.646 | 180 | 178.354 | 1 |
| Skippy | 2 | M | Australian Cattle Dog | Flowers | 4.476 | 180 | 175.524 | 2 |
| Shadow | 3 | M | Border Collie | Eyes | 6.582 | 180 | 173.418 | 2 |
| Pepper | 6 | F | Australian Cattle Dog/ Border Collie | Flowers | 1.487 | 3.737 | 2.25 | 1 |
| Shahara | 3 | F | Cocker Spaniel/ Poodle | Eyes | 10.962 | 50.255 | 39.293 | 2 |
| Islay | 2 | F | Border Collie | Flowers | 2.071 | 121.657 | 119.586 | 1 |
| Raro | 5 | F | Staffie/ Labrador | Eyes | 2.097 | 4.688 | 2.591 | 2 |
| Henry | 7 | M | Bearded Collie X | Flowers | 1.059 | 40.005 | 38.946 | 1 |
| Bronxy | 5 | F | Corgi/ Jack Russel | Eyes | 2.499 | 3.704 | 1.205 | 1 |
| Rufus | 3 | M | Cocker Spaniel | Flowers | 1.101 | 44.933 | 43.832 | 2 |
| Wilma | 4 | F | Labrador | Eyes | 2.67 | 180 | 177.33 | 2 |
| Charlie 2 | 2 | M | Miniature Schnauzer | Flowers | 1.95 | 7.885 | 5.935 | 1 |
| Desmond | 9 | M | Cairn Terrier X | Eyes | 2.196 | 35.025 | 32.829 | 1 |
| Khya | 4 | F | Swiss Shepherd | Flowers | 1.174 | 1.936 | 0.762 | 2 |
| Oliver | 7 | M | Vizsla | Eyes | 2.669 | 2.352 | -0.317 | 2 |
| Vossy | 5 | M | Labrador | Flowers | 1.654 | 3.793 | 2.139 | 1 |
| Manny | 8 | M | German Shepherd | Eyes | 4.144 | 1.93 | -2.214 | 1 |
| Hutch | 7 | M | Border Collie/ Herding Dog | Flowers | 1.904 | 180 | 178.096 | 2 |
| Mint | 9 | F | Beagle | Eyes | 1.693 | 44.553 | 42.86 | 2 |
| Lucky | 7 | M | German Spitz X | Flowers | 2.681 | 19.856 | 17.175 | 1 |
| Kia | 4 | M | Staffie X | Eyes | 1.013 | 180 | 178.987 | 1 |
| Quinta | 7 | F | Groenendael | Flowers | 2.442 | 87.236 | 84.794 | 2 |
| Dixie | 3 | F | Greyhound | Eyes | 2.74 | 18.757 | 16.017 | 2 |
| Maia | 2 | F | Groenendael | Flowers | 1.903 | 153.633 | 151.73 | 2 |
| Bruno | 2 | M | Unknown | Eyes | 4.662 | 19.588 | 14.926 | 1 |
